# Is “Circling” Behavior in Humans Related to Postural Asymmetry?

**DOI:** 10.1371/journal.pone.0043861

**Published:** 2012-09-05

**Authors:** Emma Bestaven, Etienne Guillaud, Jean-René Cazalets

**Affiliations:** 1 Université de Bordeaux, Institut de Neurosciences Cognitives et Intégratives d’Aquitaine, Unité Mixte de Recherche 5287, Bordeaux, France; 2 Centre National de la Recherche Scientifique, Institut de Neurosciences Cognitives et Intégratives d’Aquitaine, Unité Mixte de Recherche 5287, Bordeaux, France; Bielefeld University, Germany

## Abstract

In attempting to walk rectilinearly in the absence of visual landmarks, persons will gradually turn in a circle to eventually become lost. The aim of the present study was to provide insights into the possible underlying mechanisms of this behavior. For each subject (N = 15) six trajectories were monitored during blindfolded walking in a large enclosed area to suppress external cues, and ground irregularities that may elicit unexpected changes in direction. There was a substantial variability from trial to trial for a given subject and between subjects who could either veer very early or relatively late. Of the total number of trials, 50% trajectories terminated on the left side, 39% on the right side and 11% were defined as “straight”. For each subject, we established a “turning score” that reflected his/her preferential side of veering. The turning score was found to be unrelated to any evident biomechanical asymmetry or functional dominance (eye, hand…). Posturographic analysis, used to assess if there was a relationship between functional postural asymmetry and veering revealed that the mean position of the center of foot pressure during balance tests was correlated with the turning score. Finally, we established that the mean position of the center of pressure was correlated with perceived verticality assessed by a subjective verticality test. Together, our results suggest that veering is related to a “sense of straight ahead” that could be shaped by vestibular inputs.

## Introduction

People walking in a natural environment without stable external visual landmarks often get lost and come full circle. This prevalent experience has often inspired various works of literature or movies over the years, but surprisingly, there have been only a few scientific reports on this phenomenon [1], [2], [3], [4], [5], [6], [7], [8]. The most likely explanation for this “circling” behavior is that the absence of a stable distant visual cue, such as in a desert, or the presence of only local cues, such as trees in a forest, do not provide the lost subject any indication of the path he has to follow.

In the first studies that addressed this issue [1], [9], it was claimed that when walking, swimming, driving a car, boat or a plane without vision, deviation was most of the time consistent in the same direction across trials. Because he observed similar spiral-shaped paths in various organisms, from spermatozoa to blindfolded humans, Schaeffer (1928) proposed that this was evidences for an ubiquitous spiraling mechanism and that “the same mechanism is at work in man that operates in the amoeba” [9]. These pioneering studies concluded that this behavior should be related to central mechanisms since the deviation always occurred in the same direction, whatever the mode of displacement. However, Lund (1930), who was unable to reproduce Schaeffer’s results, claimed that there was a relationship between the side of deviation when walking blindfolded and inequality in the length and the strength of the legs, and he concluded that veering was only related to mechanical bias [5]. This early conclusion was also drawn in a Scientific American report in 1893, i.e., “the fact that people lost on a desert or in a forest invariably walk in a circle is due to a slight inequality in the length of the legs” [7]. The hypothesis of biomechanical asymmetries was, however, refuted in several studies [8], [10], [11], because the trajectory direction was not found to be related to relative length of legs, relative length of strides or facing direction of the head relative to that of the trunk [10], [11]. Furthermore, by causing artificial differences in leg length using soles, Souman et al. (2009) found that the trajectory orientation was not dependent on this parameter. However, none of the various explanations proposed for explaining veering behavior (differences in leg length or strength, biomechanical asymmetries, lateralization) can account for the behavior described in experimental findings, because they either suffered from methodological deficiencies in measuring veering or could not be reproduced [4].

Various studies have analyzed walking direction in blind or blindfolded subjects to find strategies that may help blind subjects to walk straighter [12], [13], [14]. For example, the discovery of a correlation between walking speed and amount of veering [12], [15], [16] has led to the recommendation that patients increase their walking speed to reduce veering (e.g. for street-crossing). Although valuable results have been collected, subjects were walking only short distances (∼10 m), similar to other previous studies, [17], [18], [19], which does not allow a full description of walking behavior. Moreover, these studies were performed in small environments, which, due to subject apprehension and anxiety, clearly modify walking parameters. Finally, instructions differed; in some protocols, subjects had to memorize the direction of a previously seen target [18], [19], [20], [21], [22], [23].

Only recently has this problem begun to be experimentally addressed for longer distances based on GPS tracking [8]. In this study, the authors analyzed long trajectories in two different natural environments: a large forest area and the Sahara desert. Furthermore, they tested the ability of people to maintain a straight walking direction while blindfolded in an outdoor experimental field. Although the work has been pioneering, valuable and interesting, it has been mainly focused on descriptive aspects. Furthermore, although natural environments, such as those used by Souman et al. [8], provide large spaces, they do not allow for the control of many parameters such as the presence of visual cues (sun, moon, distant geographical landmarks). They also fail to allow for control of other sources of sensory cues, such as wind, sun, heat or noises, that may give an indication of walking direction or perturb walking due to ground irregularities. For this reason, we have designed a protocol that allows the study of walking orientation (1) in a large enough (90 m×150 m free space) and closed environment that was protected from all external perturbations, (2) using a tracking system that allowed a step-by step follow-up and provided insights on each temporal speed cycle parameter over hundreds of meters. If, as stated by Souman, veering is not due to biomechanical asymmetries, it is necessary to consider other sources of error and determine which could be the cause of this behavior. In the present study, electromyography was used during walking to determine if there were any differences in muscle activities between the body sides. Then, we used posturography and otholitic testing to assess the subjects’ tendency to lean or to estimate verticality and correlate this with veering tendencies in walking trajectory. Altogether we confirm here that veering is not due to mechanical asymmetries but could be related to an asymmetry in sensorial inputs.

## Materials and Methods

### Participants

Fifteen subjects (see Table 1) participated in the experiment (8 males and 7 females, 20–34 years, mean 27.4 SD 4.3). None of them reported vestibular or neuromuscular deficits. Four subjects were left-handed (3 males and 1 female). The subjects gave their written informed consent and the procedures were in accordance with the ethical standards in the Declaration of Helsinki. Experiments performed in this study were specifically approved by the Direction Regionale des Affaires Sanitaires et Sociales (Authorization for Biomedical Research N°LR07 delivered on april 10 2009, available until april 10 2014) and by our local ethic committee, “le Comité de Protection des Personnes Sud Ouest et Outre Mer III” (N° 2011/38 delivered on april 27 2011).

**Table 1:** Mean (SD) of subjects characteristics and hand, leg and eye dominance.

Subjects characteristics			
	Mean (SD)	N	
**Age (y)**	27.4 (4.3)		
**Height (m)**	1.71 (0.07)		
**Weight (kg)**	66.9 (8.7)		
**Gender**		8 (Male)	7 (Female)
**Hand Dominance**		4 (Left)	11 (Right)
**Support Leg**		5 (Left)	10 (Right)
**Motor Leg**		8 (Left)	7 (Right)
**Eye Dominance**		6 (Left)	9 (Right)

### Walking Task and Trajectory Measurements

The room in which the experiment took place was part of the exhibition center in Bordeaux. It consisted of a vast closed area free of any obstacle (90 m×150 m). The participants were blindfolded (wearing a mask) and were equipped with earmuffs to suppress auditory cues. They were told to walk straight at their preferred velocity and received no other instruction than “walk straight ahead.” One experimenter followed the subject and shouted to warn the subject to stop walking when they came too close to one edge of the room. At the end of the trial, to avoid knowledge about their position, blindfolded subjects were guided to their starting position. Six trials were performed blindfolded, and one trial, a control, was performed with eyes open and no earmuffs.

Trajectories were recorded with a Total Station Trimble S6 (Trimble, USA) used to locate the position of a reflector (1-Hz frequency) with an accuracy of ±3 mm. A Total Station is a device used in topography for measuring and recording angles and distance with a distance unit. The distance unit transmits a red laser beam to the target point and calculates the distance between the transmitted and the received light. The reflector was a small prism fixed on the arch of the earmuff. The trajectories were then exported from the Trimble Station: X data were the positions on the mediolateral axis and Y data were positions on the anteroposterior axis. The straight walking direction was defined from the first two meters of each trial. Instantaneous velocity was computed as the first derivative of the trajectory. All data were processed using Matlab (MathWorks, Massachusetts).

To estimate the amount of veering, each trajectory was subsequently described by a circle passing closest to each point of the trajectory. We fitted data with the least-square method and drew a circle for each trajectory (Matlab “circlefit” routine). We chose to use the radius of the circle, which seemed to be the parameter that better characterized each trajectory. The radius was positive in sign for left turns and negative for right turns.

### Electromyography

Electrodes were placed on clean skin for recording the surface EMG of various leg muscles (tibialis anterior, TA; peroneus longus, PL; gastrocnemius lateralis, GL) and trunk muscles (erector spinae, ES) because walking along curved paths also involves trunk orientation [24]. Data were collected with the Pocket EMG (BTS, Italy) at a sampling frequency of 1 kHz.

EMG signals were rectified and smoothed using a moving average on 200 samples. For each subject and each muscle, all the data were normalized in amplitude on the basis of the signal average computed on the control trial (walking straight with eyes open). For each trial, the first EMG activation was visually detected on the TA, and EMG data were synchronized with the walking start. Areas were computed by dividing the sample cumulative sum by the number of samples. Because the periods of positive radius and negative radius varied over the trials, the total areas measured during positive curve and during negative curve in each trial were normalized (in time) by dividing these areas by their duration. All trials were averaged by subjects. Step parameters were extrapolated from EMG data and trajectory data. We counted the number of steps from muscle activity (number of right cycles and left cycles), and from the total distance traveled, we were able to calculate the mean length of the step.

### Posturography

Subjects remained in a quiet erect position for 1 minute [25], [26] on a forceplate (AMTI, USA) to record 3D forces and the position of their center of pressure (COP). They adopted a self-selected comfortable position with eyes open. Each subject’s position while standing was measured using a 3D motion capture system (Elite, BTS, Italy) with 8 cameras (100 Hz precision, ±1 mm). Reflective markers were placed bilaterally on the skin in the following locations: head of the fifth metatarsal, lateral malleolus, heel, antero-superior iliac spine (ASIS), L3, acromion and C7. Kinematic data were processed using the Biomech software (BTS, Italy) to reconstruct the marker’s positions.

The base of support (BoS) was defined as the area bounded by the markers on the fifth metatarsals and heels. The COP position was calculated in the BoS of each subject.

We checked reliability of the COP position measurement by conducting 10 successive trials on control subjects (5 males and 5 females). The 10 subjects were asked to stay in a static erect position for 30 seconds with both eyes opened or eyes closed (in a random order). A 60 second rest period during which the subject had to move from the forceplate separated each trial. We found that the variability in COP placement across trials was low and that the majority of participants had a “preferred” side (which was the same for both conditions). Only two participants placed their COP in the middle of the base of support. We averaged the standard deviation calculated for each subject and compared it to the absolute mean position of the COP (mean distance to midline). Mean distance from midline was 1 cm and mean standard deviation was 3.3 mm.

Since posture and locomotion are linked [27], maximal trunk rotations were also recorded to assess if there was any functional asymmetry in trunk rotation capabilities. Subjects turned their trunk to both sides to the maximum possible. Ten trials were recorded, five on the right side and five on the left side. The following angles were computed during static posturography and during trunk rotations: shoulders/ASIS in the frontal and horizontal planes, shoulders/vertical, and ASIS/vertical (α1, α2, α3 and α4). Finally, leg length from the ASIS to the internal malleolus and leading leg dominance were collected.

### Subjective Visual Vertical

The subjective visual vertical (SVV) was assessed by using a test for otolithic function (Synapsys Europe, France) in the dark. A bar was projected on the wall with a video projector and the subject was asked to place this bar in a vertical position. As specified in the Synapsys protocol, the bar was initially placed in different positions between trials. The experimenter moved the bar until the subject told him to stop [28] and recorded the angular difference between the actual vertical and the perceived vertical. Three trials were recorded and mean angle value was calculated.

As for the COP measurement, we checked the reliability of the SVV measurement by performing 10 successive trials on a control group consisting of 5 males and 5 females. We averaged the standard deviation calculated for each subject and compared it to the absolute mean angle value. The absolute mean value was 0.77° and mean standard deviation was 0.75°.

### Statistics

Statistical analysis was performed using IBM SPSS Statistics 19 (IBM Corporation, USA). Results are presented as mean and standard deviation (±SD). The strengths of the relationships were determined using the Pearson’s correlation coefficient (R). However, when a variable had a low number of values, non-parametric Spearman’s rank correlation coefficient (Rho) was used. Non-parametric tests were also used to compare group means (Mann-Whitney test) and to compare parameters across trials (Friedman test). Results were considered statistically significant for P<0.05. For the multiple correlations between the turning score, the position of the COP and the SVV, a correction was applied to significance level with the Holm-Bonferroni method.

## Results

### Walking Trajectories

**Figure 1:**
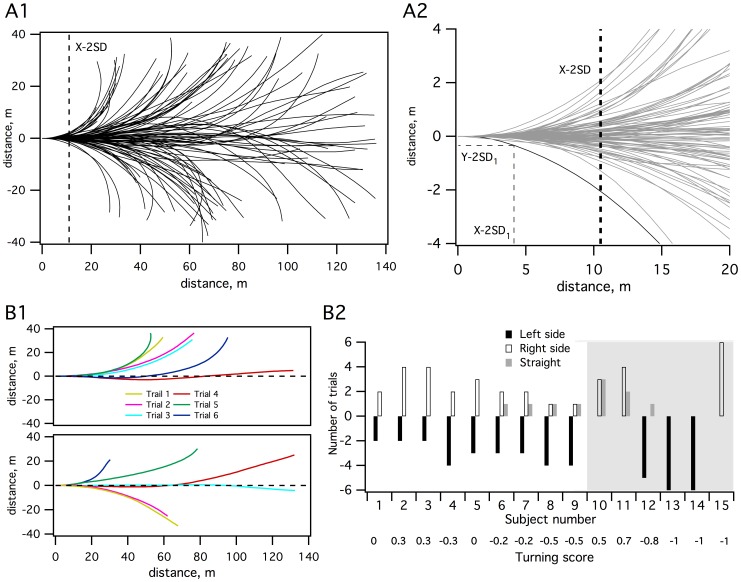
Trajectories during blindfold walking. **A1:** Graphical representation in the horizontal plane of all recorded trajectories (n = 88) in the whole area. Due to technical reasons, one of the participants only performed 4 trials instead of the 6. The x-axis represents the straight ahead direction. X-2SD (vertical dotted line) is the mean position value on the x-axis at which the deviation occurs for all trajectories. **A2:** Enlarged view of the first 20 m for all trajectories. Y-2SD_1_ and X-2SD_1_ are the positions on the y-axis and on the x-axis respectively at which the deviation occurs for one example trajectory (black curve). X-2SD (vertical dotted line) is the mean position on the x-axis at which the deviation occurs for all trajectories. **B1:** Walking trajectories of two subjects; the top graph shows a consistent subject that veers on the same side (left side), the bottom graph shows an inconsistent subject that veers on both sides. The dotted line indicates the straight ahead direction. **B2:** “Turning score”: number of trials that veer to the left, the right and straight ahead (black, white and grey) for all subjects (n = 15). The turning score is annotated on the bottom line; subjects who always turned on the left side scored −1 while subjects who always turned on the right side scored +1. Subjects who had an equal number of trials on both sides scored 0. The grey area overlaps consistent subjects.

All of the trajectories (n = 88) that the 15 subjects performed within the experimental area are plotted in Figure 1A, using the starting point as the zero reference. Due to technical reasons, one of the participants only performed 4 trials instead of the normal 6. One striking characteristic is the wide variability of the trajectories; some of them reached the edge of the area (Y axis) in less than 30 m (in the X direction) while other trajectories still remained straight at 140 m. The majority (50%) of the trajectories ended on the left side, 39% on the right side and 11% were defined as “straight”. By convention, we defined straight trajectories as trials for which we found a final deviation of less than 10% of the total trajectory length. We arbitrarily considered this cut-off to be superposed by a circle radius >300 m. These parameters helped us to define rectilinear trajectories. The total distance traveled ranged from 24 m to 143 m (mean 88±32.2 m). Figure 1A2 presents an enlarged view of the first 20 m for all the trajectories. An index of deviation was calculated based on the standard deviation of Y displacement measured during the control trial with open eyes (Y-SD, mean 8 cm). A trajectory was considered to deviate from the straight direction when the Y value was greater than 2 *Y-SD (Y-2SD). We then calculated, for each subject’s trajectory, the position on the X axis at which the deviation occurred (X-2SD Fig. 1A2). The dotted line (Fig. 1A2) indicates mean X-2SD (mean value 10.5±6.5 m). Some subjects veered very early in a trial (3 m), while others walked straight for more than 30 m.

Among subjects, two behaviors were observed: (1) subjects that always veered on the same side or walked straight forward (consistent subjects, CS n = 6, top panel Fig. 1B1); (2) subjects that veered on one side or the other or walked straight forward (inconsistent subjects, IS, n = 9, bottom panel Fig. 1B1). Figure 1B1 also reveals that there was an important variability from trial to trial for one given subject that performed either very short or very long pathways. Furthermore, there was no learning effect on the distance traveled: there was no significant difference in the length of the trajectory from trial to trial (P = 0.9).

For each subject, we established a “turning score” (Fig. 1B2), that reflects his/her propensity to preferentially veer to one side or the other. Subjects who always turned to the left side scored −1 while subjects who always turned to the right side scored +1. Subjects who had an equal number of trials on both sides scored 0. Using Mann-Whitney tests, we found that the turning score was not influenced by hand, foot or eye dominance (P = 0.9, P = 0.6, P = 0.5), or by gender (P = 0.7). It was not related to a difference in leg length (Rho = 0.077, P = 0.4) or to a functional imbalance in trunk rotations (Rho = 0.075, P = 0.4; see Materials and Methods for these parameter assessments).

**Figure 2:**
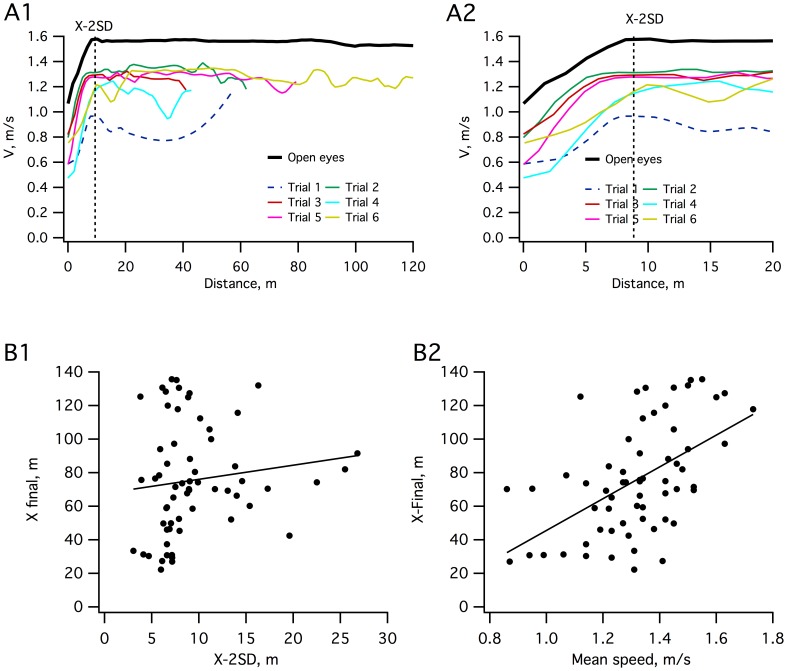
Speed and walking trajectories. **A:** One representative subject showing instantaneous velocity for the 6 trials performed with the eyes closed and the control trial performed with open eyes (**A1**). **A2:** An enlarged view of the speed profile during the first 25 m (same subject shown in A1). X-2SD (vertical dotted line) indicates the mean position value on the x-axis at which the deviation occurs (see Figure 1A). **B1:** Plot of the final position on the x-axis (X final) versus the position value on the x-axis at which deviation occurs (X-2SD) for all trajectories (n = 88). **B2:** Plot of the final position on the x-axis (X final) versus the mean speed.

For the second step, we measured various gait parameters for all subjects (Table 2). For all gait parameters, no significant differences were found between the consistent and inconsistent groups. One critical parameter measured was the walking speed of the subjects, because it likely reflected the subject’s confidence when blindfolded. In fact, as shown in Figure 2A, this revealed some interesting results. First, all subjects clearly exhibited a different speed profile for the first trial (mean 1.22±0.2 m/s, P<0.01), compared to the following trials, likely due to apprehension of the first trial blindfolded. For this reason, the first trials were excluded from all analyses. Second, the mean speed of the trial completed with eyes open (1.59±0.15 m/s) was slightly higher than the mean speed of blindfold trials (1.32±0.18 m/s, P<0.002). Finally, as observed in Figure 2A2 which presents an enlarged view of the speed profile during the first 25 meters, velocity progressively increased from the beginning until it reached a steady value at about 8 meters. Speeds in the open eye trial exhibited the same profile as in the other trials.

**Table 2:** Mean (SD) gait parameters during blindfolded walking task.

Gait parameters	Mean (SD)		
	Consistent Subjects	Inconsistent Subjects	P
**Path length (m)**	83.8 (27.1)	95.6 (13.4)	0.195
**Step length (m)**	0.68 (0.07)	0.69 (0.04)	0.859
**Velocity (m/s)**	1.27 (0.2)	1.37 (0.11)	0.376
**Number of steps/100 m**	149.2 (15.7)	145.5 (8)	0.897

We then determined if the shape of the trajectory at the beginning of the trial could predict the final position. As indicated above, we defined the onset of the deviation (Y-2SD) and the X position at this point (X-2SD) for all trajectories. We therefore tested if there was a correlation between X-2SD and the final position on the X axis. Figure 2B1 shows there was no significant correlation (R = 0.24, P = 0.07), indicating that the position at about 10 meters after the beginning of the trial could not correctly predict the direction of the whole trajectory and the final position. Moreover, X-2SD was not correlated with velocity at the onset of deviation (R = 0.152, P = 0.3). On the contrary, as shown in Fig. 2B2, the final position was strongly correlated with the mean velocity for all trials (R = 0.586, P<0.01). The correlation between speed and distance was not due to within- or between-subject influences since we found comparable values when considering the mean value of these variables for each subject. (R = 0.049, P = 0.9 for mean X-2SD and mean final position on the X axis; R = 0.134, P = 0.6 for mean X-2SD and mean velocity at the onset of deviation; R = 0.789, P<0.01 for mean final position and mean velocity). Therefore, the faster the subject walked, the longer the trajectory (Fig. 2B2). As a steady speed is only reached after 8 m (Fig. 2A2), and as there is an interaction between velocity and deviation, measurements of the deviations on 10 m pathways were unlikely to provide relevant insights on this behavior (see Discussion).

**Figure 3:**
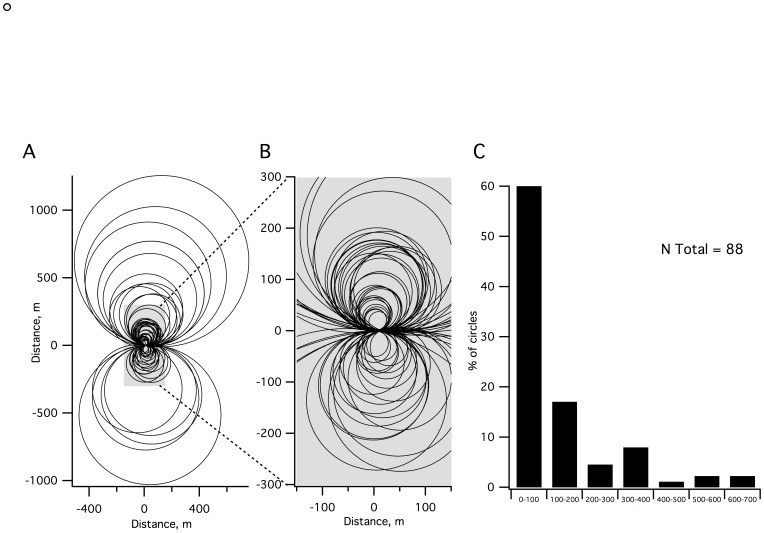
Radius circle of the trajectory. **A:** Circles fitted for all trajectories (**A**) and enlarged view for the circles with radius <300 m (**B**). Over 80% of the trials are displayed in this view. **C:** Distribution of trials according to their radius circle. Seven classes are represented: <100 m, 100–200 m, 200–300 m, 300–400 m, 400–500 m, 500–600 m and 600–700 m. Approximately 80% of the trials belong to the first three classes.

The radius of the circle that best described the trajectory (see Materials and Methods) is an interesting parameter to measure veering because it reflects the geometric shape of the trajectory. As illustrated in Figure 3A, which shows the circles for all trajectories, absolute radius varied between trials and ranged from 19 m to 658 m (mean 150±155.1 m). However, 80% of trials had a radius less than 300 m (Fig. 3A and 3B). As a measure of the deviation, radius was indeed correlated with the final value on the X axis (R = 0.882, P<0.01) and significantly correlated with the mean walking speed (R = 0.456, P<0.01).

One possible explanation of veering could be the existence of an asymmetry in motor output. To test this possibility, we monitored the EMG activity of various muscles (see Materials and Methods) to detect a possible right/left difference. We could not find any significant changes in leg or trunk muscle recorded activity during veering under our experimental condition when compared to straight walking in the open eye condition.

### Posturography

**Figure 4:**
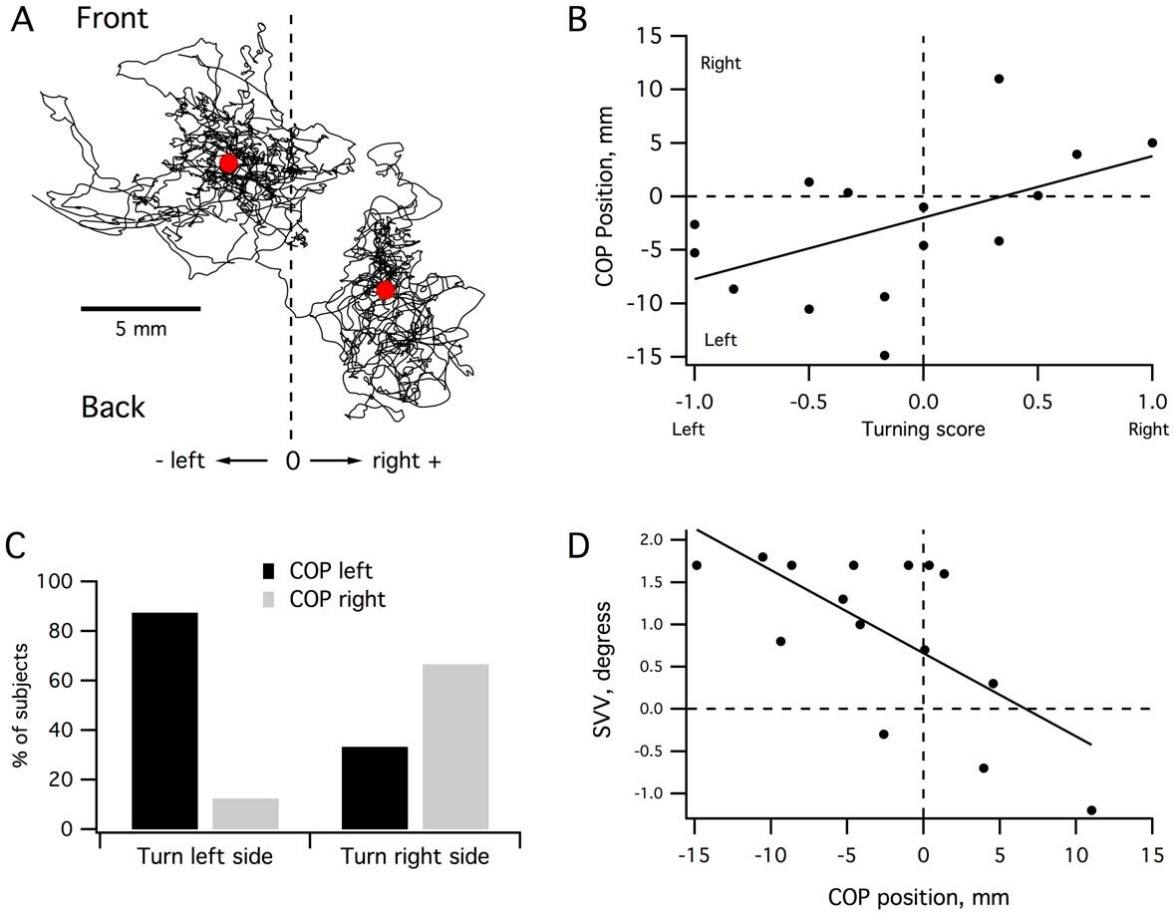
Center of pressure (COP) during posturographic tests. **A:** Stabilograms for two representative subjects. The vertical dotted line represents the midline of the base of support that is determined from the position of the feet. The black curves represent the COP position during a 1-minute balance test. The white diamond indicates the mean position of the COP trajectory. **B.** Relationships between posturographic parameters and walking trajectories: correlation analysis of mean COP position versus turning score for all subjects. **C.** Percentage of subjects with the COP on the left side and the COP on the right side during the one minute balance test. **D.** Relationships between posturographic parameters and subjective visual vertical.

Posturographic data were used to assess if there was a relationship between functional postural asymmetry and veering. The mean COP position during the postural test with eyes open was calculated (Fig. 4A) to detect any postural asymmetry. We found that each subject preferentially placed the COP on one side of the base of support (BoS): 9 subjects on the left side and 6 on the right side. To determine if there was a relationship between posture and deviation during blind locomotion, we correlated mean COP position (COP distance from midline) and turning score (Fig. 4B). We found a significant correlation between these two parameters (Rho = 0.531, P<0.025). Indeed, over 80% of subjects who turned to the left for the task of locomotion placed the COP on the left, while 70% of subjects who had turned to the right moved their COP to the right during the posture test (Fig. 4C). This correlation increased if we consider only the Consistent subjects (Rho = 0.812, P<0.025).

**Figure 5:**
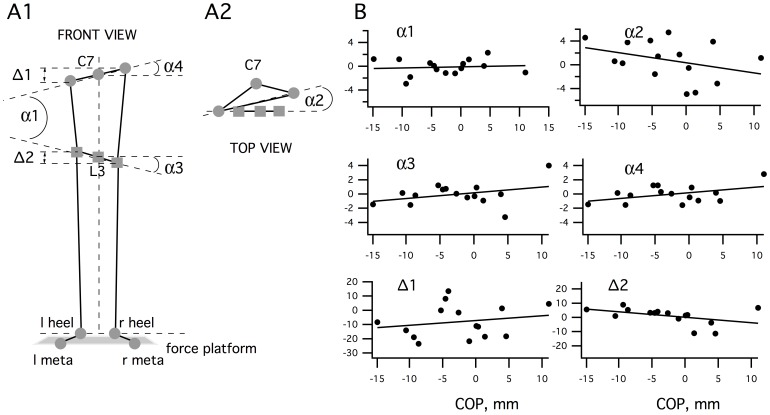
Kinematic analysis during static posture. **A:** Schematic frontal view of the subject (**A1**). Relative position of the pelvis and the trunk in the frontal plane (α1), the angle between the pelvis and the horizontal axis (α3), the angle between the trunk and the horizontal axis (α4), the height position between right and left shoulders (Δ1) and the height position between the right and left ASIS (Δ2); Schematic horizontal view of the trunk (**A2**). Relative position of the pelvis and the trunk in the horizontal plane (α2). **B:** Correlation analysis between kinematic parameters and mean position of the COP during a 1-minute balance test.

We used kinematic measurements of various body coordinates (Fig. 5A) to see if the asymmetry observed in COP position could be related to anatomical asymmetries in the upright position during the postural test. For this purpose, we analyzed the various angles and corporal segments as indicated in Figures 5A1 and A2. We did not find significant relationships between the position of the COP and any of the measured parameters (Fig. 5B): (1) the relative position of the pelvis from the trunk in the sagittal plane (α1; Rho = 0.05, P = 0.9); (2) the relative position of the pelvis from the trunk in the frontal plane (α2; Rho = −0.361, P = 0.2); (3) the angle between the pelvis and the vertical axis (α3; Rho = 0.1, P = 0.7); (4) the angle between the trunk and the vertical axis (α4; Rho = 0.204, P = 0.5); (5) the difference between position of the right shoulder and the left shoulder on the vertical axis (Δ1; Rho = 0.150, P = 0.6); (6) the difference between the position of the right ASIS and the left ASIS on the vertical axis (Δ2; Rho = −0.496, P = 0.6). These results suggest that the shift of COP relative to the base of support does not rely on mechanical asymmetries.

Subjective visual vertical cues contribute to the development of an internal model of verticality that is useful in balance control [28]. Therefore, we checked to see if the shift of the COP could be related to a misperception of verticality. For this purpose, all subjects passed a subjective visual vertical (SVV) test (see Materials and Methods). Results of the SVV test ranged from −1.2° to 1.8° (mean 0.92±0.8°) and indicated that no participants had vestibular impairments (pathologic threshold 3°) [4]. We then compared the mean position of the COP and mean deviation of the subjective visual vertical test and found that these two parameters were strongly correlated (Rho = −0.626, P = 0.01; Fig. 4D). This negative correlation indicated that subjects who shifted their COP to the left side were those with SVV shifted on the right side while subjects who shifted their COP to the right side had a SVV shifted on the left side. In other words, a postural imbalance on the left side could be related to a right imbalance in the perception of verticality, and vice versa. However, we did not find significant correlation between the SVV and the turning score (Rho = −0.437, P = 0.104).

## Discussion

In the present study, we provided detailed characteristics of the circling behavior in the absence of vision using trajectory tracking systems in a very large closed environment. Furthermore, we performed electromyographic, kinetic, and kinematic analyses to establish the underlying mechanisms of this behavior. In addition to providing information on the “circling behavior”, the accuracy of our measurements brings new interesting results on the initial part of veering (first 10 m) and allowed us to highlight several inconsistencies in previous studies on veering when walking blindfolded. Our results indicate that veering is not due to anatomical or biomechanical asymmetries rather that it could be related to postural performance.

### Experimental Protocol

A crucial point, and one which is likely key to the discrepancies observed between studies [4], is the question of whether the experimental design used to test circling behavior is reliable in addressing this phenomenon. We believe that the protocol that we used here alleviates most of the experimental bias that could have occurred in previous studies. First of all, one critical feature of our protocol was that the subjects moved in a closed space in which external sensory cues that may give indications on walking direction, such as wind, sun, heat, or noises, or ground irregularities that may unbalance the subject and perturb walking, were virtually abolished.

Another important point was the room size. In fact, most of the previous studies were performed in relatively small rooms using short distances (between 10 to 20 m) [14], [17], [18], [19], [22], [23], [29]. However, our results demonstrate that walking such short distances does not allow for the collection of reliable data for describing this behavior. We found that the onset of deviation occurs as late as 10 m on average but that this value could rise up to 30 m. However, we also established that a steady speed is only reached at about 8 m (Fig. 2A2), indicating that data collected on short distances is unreliable. Furthermore, thanks to our measurement technique, we were able to calculate the step by step velocity, and we measured a significant negative correlation between walking speed and the radius circle. These results are consistent with previous reports [12], [16], in which a relationship between walking speed and veering was also found. We determined, however, that the walking speed of the first trial for each subject was significantly different from the other trials and that, in the absence of visual and auditory cues, subjects always walked at a slightly lower speed than with open eyes (Fig. 2A). This is likely due to the subject’s apprehension, even though in our testing conditions, this apprehension should have been minimized due to the very large space available. Finally, we established that there was no relationship between the position at 10 m and the final position (Fig. 2B1), therefore making it difficult to extrapolate trajectories from a 10 m walking distance as was previously done [14]. Analyzing trajectories in outdoor areas, Souman et al. (2009) found that blindfolded subjects walked in circles of comparable diameter to those observed in our experimental conditions (Fig. 3A). Therefore, our protocol quite accurately reproduces the behavior observed in these large-scale open spaces while allowing us to more systematically test the onset of deviation in the absence of any perturbations. Altogether, these results further emphasize the requirement for performing these types of experiments in a very large space free of obstacles and raise suspicions regarding previous conclusions from studies using other testing parameters.

### Are the Characteristics of Circling Behavior Related to Biomechanical Asymmetries?

Studies undertaken to explain veering have been conducted regularly since the XIX century. In their review, however, Guth and Laduke [4] pointed out the contradictory conclusions drawn by these early studies. Therefore, the first issue we have addressed in this study was to accurately describe the trajectory performed by subjects in the absence of visual and auditory cues and to test whether or not subjects always veered in the same direction in the absence of visual landmarks.

In the present study, we did not establish any relationship between the turning score and the various biomechanical parameters that we measured during standing position (Fig. 5). Furthermore, it is likely that an asymmetry such as a difference in leg length or strength would lead to more systematic trajectory orientation which does not appear to be the case. Finally, several types of dominance such as hand, leg or eye dominances have been proposed to explain trajectory orientation [5], [30]. However, in accordance with several other studies [1], [8], [29], we did not find any correlation between these parameters and veering.

In our study, we did not find any significant preference for right or left orientation when considering the overall trials (N = 88) performed by all the subjects, as also previously reported [1], [8], [19]. However, we were able to define two groups of subjects, consistent subjects veering always in the same side and inconsistent subjects alternating their orientation from trial to trial. Such behaviors were also reported as “homotropic” and “heterotropic” [1], [17]. Unlike Cratty and Williams (1966), who reported that 80% of the subjects exhibited an homotropic behavior, we found here that consistent subjects only represented 40% of the sample [12]. This may be attributable to the fact that Cratty and Williams defined homotropic subjects as people who veered in the same direction in only three out of four trials [12], while in our study, subjects were considered as homotropic when they always veered in the same direction for all six trials.

The EMG activity of various muscles was recorded to detect possible asymmetries in motor output. We did not find any difference between the right and left sides in leg and trunk muscle activity during veering under our experimental conditions when compared to straight walking in the open eye condition. This may be attributable to the fact that subtle changes could not be detected by our analysis method or that curved trajectories performed by subjects were too smooth, hence any changes were too small to be detected. As reported in a previous study, only subtle changes were observed on more pronounced curves [31].

### Is Circling Behavior Related to Postural Performances?

During blindfold walking, our sense of progression relies on the processing of internally generated self-motion signals or idiothetic information [32] including proprioceptive, vestibular and motor efference copy. In addition, motion perception depends on internal models. Most of the studies that have addressed walking in blindfolded subjects, however, have been focused on path-integration or orientation towards a visual target presented prior to initiating movement, or reproduction of a recently performed route [33], [34], [35], [36]. The task performed in the present study was, however, of a different nature because it did not rely on any spatial memory ability but rather on a subjective “sense of straight ahead” [8].

Because our study suppressed or limited most of the extrinsic hints that could have influenced walking direction (wind, sun, heat, ground slope etc.), the only sensory inputs on which the subjects could rely were their vestibular and proprioceptive inputs to get information on their position in space. Our posturographic data reveal that the turning score is correlated to the mean position of the COP (Fig. 4B) and that the COP itself is correlated with SVV. Although the test used in our study does not allow discrimination between vestibular and proprioceptive contributions to the slight shift in COP position observed between subjects, vestibular inputs are likely involved. The vestibular system contributes to balance control during locomotion [37] and various studies have investigated the involvement of the vestibular system in balance, locomotion, and orientation in patients with vestibular impairment [38]. They reported: (i) a shift of the COP towards the side of the lesion [39], [40], [41], (ii) a deviation of the walking trajectory towards the side of the lesion [42], [43], and (iii) a tilt of the perceived visual vertical towards the side of the lesion [44], [45]. This pattern was, however, dependent on the sensory cues available [46]; when some visual cues were provided, the tilt of perceived visual vertical decreased and the COP shifted towards the intact side [39]. The negative correlation between the mean position of the COP and the SVV that we found is consistent with the existence of this postural reversal. In addition, and consistent to our results (Fig. 2B2), an increase in walking speed decreases the trajectory deviation in vestibular patients [42]. Comparable results are also observed when a galvanic vestibular stimulation is applied in healthy subjects [47]. Altogether, this suggests that the veering during blindfolded walking as reported here could be related to slight asymmetries from vestibular inputs that remain non pathological.

It would have been relevant to check whether there was some covariation in the variances of turning and COP placement, which in turn could allow us to establish a causal link between the two parameters. Indeed, a person with a high variability in postural control (across trials) might also be more variable in his/her veering tendency while walking blindfolded. However, our protocol did not allow us to assess this relationship because only a single trial in posturography could be recorded. Clearly, our results do not support a direct causal relationship between parameters, but as previously discussed, the involvement of the vestibular system in balance control could be postulated.

Even if veering originates from a slight vestibular asymmetry, this behavior is probably not mediated by a low level sensori-motor process. In fact, a vestibular asymmetry would result in a drift in the sensori-motor system, which, in turn, would generate a systematic veering in the same direction for one subject (according to the vestibular asymmetry). For many subjects (Fig. 1) the direction changes from trial to trial, suggesting that veering does not directly depend on a sensori-motor drift. Recent data on internal models of spatial representation [39], [48] and the sense of verticality [28] and its neuronal substrates [49], suggest that vestibular signals are involved in building body representation in extrapersonal space representation. As previously mentioned, we did not address an extrapersonal space representation but rather a “sense of straight ahead” [8], as the task did not require a subject mapping in an allocentric reference frame. As described by other authors [50], such a process could rely on vestibular inputs (and other sensory inputs) to compute an internal representation of the straight-ahead direction. These latter authors also concluded that this hypothetical mechanism for straight ahead could be extended to gait deviations during normal walking in the presence of vestibular tone asymmetry. Although our results do not support a direct causal relationship between turning score, COP position and SVV, the correlations we found may be explained by such a mechanism.

## References

[1] Carroll T, McAvoy WH (1929) Spiral tendency in blind flying. National Advisory Committe for Aeronautics 314.

[2] Guldberg FO (1897) Circular motion as a basic animal motion, its origin, the phenomenon, and its meaning. Zeitschriftfur Biologie 35: 419–458.

[3] Guldberg GA (1896) Abot the morphological and functional asymmetry of the limbs of humans and higher level vertebrates. Biologisches Zentralblatt 16: 806–813.

[4] Guth D, Laduke R (1994) The Veering Tendency of Blind Pedestrians - an Analysis of the Problem and Literature-Review. J Vis Impair Blind 88: 391–400.

[5] Lund FH (1930) Physical asymmetries and disorientation. Am J Psychol 42: 51–62.

[6] Schaeffer AA (1928) Spiral movement in amebas. Anat Rec 34: 115.

[7] ScientificAmerican (1893) Why lost people walk in circles.

[8] Souman JL, Frissen I, Sreenivasa MN, Ernst MO (2009) Walking Straight into Circles. Curr Biol 19: 1538–1542. 19699093 10.1016/j.cub.2009.07.053

[9] Schaeffer AA (1928) Spiral movement in man. J Morphol Physiol 45: 293–370.

[10] Cratty BJ (1965) Perceptual thresholds of non-visual locomotion (Part 1). Los Angeles: University of California.

[11] Cratty BJ (1967) The perception of gradient and the veering tendency while walking without vision. American Foundation for the Blind Research Bulletin 14: 13–51.

[12] Cratty BJ, Williams HG (1966) Perceptual thresholds of non-visual locomotion (Part 2). Los Angeles: University of California.

[13] Guth D, Laduke R (1995) Veering by Blind Pedestrians - Individual-Differences and Their Implications for Instruction. J Vis Impair Blind 89: 28–37.

[14] Kallie CS, Schrater PR, Legge GE (2007) Variability in stepping direction explains the veering behavior of blind walkers. J Exp Psychol - Hum Percept 33: 183–200. 17311487 10.1037/0096-1523.33.1.183 PMC2259118

[15] Cicinelli J (1989) Veer as a function of preview and walking speed. Santa Barbara: University of California.

[16] Klatzky RL, Loomis JM, Golledge RG, Cicinelli JG, Doherty S, et al (1990) Acquisition of Route and Survey Knowledge in the Absence of Vision. J Mot Behav 22: 19–43. 15111279 10.1080/00222895.1990.10735500

[17] Boyadjian A, Marin L, Danion F (1999) Veering in human locomotion: the role of the effectors. Neurosci Lett 265: 21–24. 10327196 10.1016/s0304-3940(99)00198-6

[18] Courtine G, Schieppati M (2003) Human walking along a curved path. I. Body trajectory, segment orientation and the effect of vision. Eur J Neurosci 18: 177–190. 12859351 10.1046/j.1460-9568.2003.02736.x

[19] Mohr C, Brugger R, Bracha HS, Landis T, Viaud-Delmon I (2004) Human side preferences in three different whole-body movement tasks. Behav Brain Res 151: 321–326. 15084448 10.1016/j.bbr.2003.09.006

[20] Amorim MA, Glasauer S, Corpinot K, Berthoz A (1997) Updating an object’s orientation and location during nonvisual navigation: A comparison between two processing modes. Percept Psychophys 59: 404–418. 9136270 10.3758/bf03211907

[21] Glasauer S, Amorim MA, Viaud-Delmon I, Berthoz A (2002) Differential effects of labyrinthine dysfunction on distance and direction during blindfolded walking of a triangular path. Exp Brain Res 145: 489–497. 12172660 10.1007/s00221-002-1146-1

[22] Takei Y, Grasso R, Amorim MA, Berthoz A (1997) Circular trajectory formation during blind locomotion: A test for path integration and motor memory. Exp Brain Res 115: 361–368. 9224864 10.1007/pl00005705

[23] Vieilledent S, Kosslyn SM, Berthoz A, Giraudo MD (2003) Does mental simulation of following a path improve navigation performance without vision? Cogn Brain Res 16: 238–249. 10.1016/s0926-6410(02)00279-3 12668233

[24] Grasso R, Prevost P, Ivanenko YP, Berthoz A (1998) Eye-head coordination for the steering of locomotion in humans: an anticipatory synergy. Neurosci Lett 253: 115–118. 9774163 10.1016/s0304-3940(98)00625-9

[25] Lafond D, Duarte M, Prince F (2004) Comparison of three methods to estimate the center of mass during balance assessment. J Biomech 37: 1421–1426. 15275850 10.1016/S0021-9290(03)00251-3

[26] Duarte M, Freitas SM (2010) Revision of posturography based on force plate for balance evaluation. Rev Bras Fisioter 14: 183–192. 20730361

[27] Massion J, Alexandrov A, Frolov A (2004) Why and how are posture and movement coordinated? Brain Mechanisms for the Integration of Posture and Movement. Amsterdam: Elsevier Science Bv. 13–27. 10.1016/S0079-6123(03)43002-1 14653147

[28] Barra J, Marquer A, Joassin R, Reymond C, Metge L, et al (2010) Humans use internal models to construct and update a sense of verticality. Brain 133: 3552–3563. 21097492 10.1093/brain/awq311

[29] Mohr C, Lievesley A (2007) Test-retest stability of an experimental measure of human turning behaviour in right-handers, mixed-handers, and left-handers. Laterality 12: 172–190. 17365633 10.1080/13576500601051580

[30] Sadeghi H, Allard P, Prince F, Labelle H (2000) Symmetry and limb dominance in able-bodied gait: a review. Gait Posture 12: 34–45. 10996295 10.1016/s0966-6362(00)00070-9

[31] Courtine G, Schieppati M (2003) Human walking along a curved path. II. Gait features and EMG patterns. Eur J Neurosci 18: 191–205. 12859352 10.1046/j.1460-9568.2003.02737.x

[32] Mittelstaedt ML, Mittelstaedt H (2001) Idiothetic navigation in humans: estimation of path length. Exp Brain Res 139: 318–332. 11545471 10.1007/s002210100735

[33] Berthoz A, Viaud-Delmon I (1999) Multisensory integration in spatial orientation. Curr Opin Neurobiol 9: 708–712. 10607650 10.1016/s0959-4388(99)00041-0

[34] Fetsch CR, Deangelis GC, Angelaki DE (2010) Visual-vestibular cue integration for heading perception: applications of optimal cue integration theory. Eur J Neurosci 31: 1721–1729. 20584175 10.1111/j.1460-9568.2010.07207.x PMC3108057

[35] Lee SA, Spelke ES (2010) Two systems of spatial representation underlying navigation. Exp Brain Res 206: 179–188. 20614214 10.1007/s00221-010-2349-5 PMC3129622

[36] Philbeck JW, Klatzky RL, Behrmann M, Loomis JM, Goodridge J (2001) Active control of locomotion facilitates nonvisual navigation. J Exp Psychol Hum Percept Perform 27: 141–153. 11248929

[37] Iles JF, Baderin R, Tanner R, Simon A (2007) Human standing and walking: comparison of the effects of stimulation of the vestibular system. Exp Brain Res 178: 151–166. 17031681 10.1007/s00221-006-0721-2

[38] Borel L, Lopez C, Peruch P, Lacour M (2008) Vestibular syndrome: a change in internal spatial representation. Neurophysiol Clin 38: 375–389. 19026958 10.1016/j.neucli.2008.09.002

[39] Borel L, Harlay F, Magnan J, Lacour M (2001) How changes in vestibular and visual reference frames combine to modify body orientation in space. Neuroreport 12: 3137–3141. 11568652 10.1097/00001756-200110080-00031

[40] Lacour M (2006) Restoration of vestibular function: basic aspects and practical advances for rehabilitation. Curr Med Res and Opin 22: 1651–1659. 16968568 10.1185/030079906X115694

[41] Takemori S, Ida M, Umezu H (1985) Vestibular Training after Sudden Loss of Vestibular Functions. Orl 47: 76–83. 3872435 10.1159/000275748

[42] Borel L, Harlay F, Lopez C, Magnan J, Chays A, et al (2004) Walking performance of vestibular-defective patients before and after unilateral vestibular neurotomy. Behavioural Brain Res 150: 191–200. 10.1016/S0166-4328(03)00257-2 15033292

[43] Cohen HS (2000) Vestibular disorders and impaired path integration along a linear trajectory. J Vestibul Res-Equil 10: 7–15. 10798829

[44] Friedman G (1970) Judgement of Visual Vertical and Horizontal with Peripheral and Central Vestibular Lesions. Brain 93: 313–&. 10.1093/brain/93.2.313 5310320

[45] Vibert D, Hausler R (2000) Long-term evolution of subjective visual vertical after vestibular neurectomy and labyrinthectomy. Acta Oto-Laryngologica 120: 620–622. 11039872 10.1080/000164800750000432

[46] Borel L, Lopez C, Peruch P, Lacour M (2008) Vestibular syndrome: A change in internal spatial representation. Clin Neurophysiol 38: 375–389. 10.1016/j.neucli.2008.09.002 19026958

[47] Fitzpatrick RC, Wardman DL, Taylor JL (1999) Effects of galvanic vestibular stimulation during human walking. J Physiol (Lond) 517: 931–939. 10358131 10.1111/j.1469-7793.1999.0931s.x PMC2269389

[48] Ivanenko YP, Dominici N, Daprati E, Nico D, Cappellini G, et al (2011) Locomotor body scheme. Hum Mov Sci 30: 341–351. 21453667 10.1016/j.humov.2010.04.001

[49] Perennou DA, Mazibrada G, Chauvineau V, Greenwood R, Rothwell J, et al (2008) Lateropulsion, pushing and verticality perception in hemisphere stroke: a causal relationship? Brain 131: 2401–2413. 18678565 10.1093/brain/awn170

[50] Marques B, Colombo G, Muller R, Dursteler MR, Dietz V, et al (2007) Influence of vestibular and visual stimulation on split-belt walking. Exp Brain Res 183: 457–463. 17665177 10.1007/s00221-007-1063-4

